# Cross-Sectional Observational Study on Association of Polypharmacy With Health-Related Quality of Life in Patients With Hypertension

**DOI:** 10.7759/cureus.30361

**Published:** 2022-10-16

**Authors:** Tanisha Paramba, Sarju Zilate

**Affiliations:** 1 Pharmacology, Jawaharlal Nehru Medical College, Datta Meghe Institute of Medical Sciences, Wardha, IND

**Keywords:** sf-36, comorbidities, polypharmacy, health-related quality of life, hypertension

## Abstract

Introduction

Hypertension is one of the major co-morbidities affecting older Indians, though current trends show that it is increasingly being diagnosed in younger adults as well. In elderly members of the population, it has been shown to be associated with other co-morbidities, making its management difficult. Among the issues that have arisen with its treatment is the increased prevalence of polypharmacy. Thus, there is a need to identify the issues arising from this increase in medications. In particular, the patient’s health-related quality of life (HRQoL) can be assessed and interpreted to ensure only appropriate polypharmacy is practiced.

Methods

The adjusted Research and Development (RAND) 36-Item Health Survey 1.0 for health-related quality of life was sent to a consecutive sampling of 100 hypertensive patients at a rural tertiary care hospital in Wardha District. They were all clinically diagnosed with hypertension and had been prescribed allopathic medication for the same. They were instructed to answer all the questions to the best of their abilities, and each question was then scored from 0 to 100. In addition, they were given questions regarding their age, sociodemographic details, number of medications and frequency of dosage, and additional co-morbidities. The independent variable, i.e., the number of medications (polypharmacy), was then compared to the physical and mental scores they received on the 36-Item Short Form survey (SF-36) to see if there was an association between the two.

Result

The patients with hypertension that satisfied the criteria for polypharmacy scored lower in the Physical Component Score (PCS) of the HRQoL with a mean difference of 10.4 points. This is a significant value, and when studied in a multivariate linear regression model, controlling for the covariates mentioned above, indicated a statistically significant and negative association between the number of medications and adjusted PCS scores (β = −5.437, p<0.05, 95% CI −8.392 to −2.482). In regards to the Mental Component Score (MCS) of the HRQoL, a difference of 3.72 points was observed unadjusted and, upon controlling for covariates, it was found to be statistically significant (β = −2.825, p<0.05, 95% CI −5.300 to −0.351).

Conclusion

There is a negative correlation between HRQoL and polypharmacy in hypertensive patients. This is especially evident in the physical aspect, as can be inferred from the Physical Component Scores attained in the study. A smaller but still significant negative correlation is seen in the mental component as well. Hence, a change of policy is indicated to idealize prescriptions and physicians must be vigilant about inappropriate polypharmacy.

## Introduction

The complications of hypertension remained the leading killer globally in 2020, with an estimated death toll of more than 10 million worldwide. The majority of hypertension management guidelines recommend that the diagnosis of hypertension be made at or above 140 mm Hg for systolic blood pressure, above 90 mm Hg for diastolic blood pressure, or both [1,2]. Due to the gradual development of stiffness in arterial walls and modifications in the compliance of arteries, hypertension has a disproportionate effect on elderly people. Evidence indicates that this is primarily due to the environmental and lifestyle changes that the modern world has afforded us [3]. Hypertension is very much on the rise, especially in developing countries with an income at the middle and lower end of the scale [4]. In 2019, it was reported that in India, approximately 29.8% of people suffered from increased blood pressure. The gap between rural and urban afflicted was also found to be narrowing as diet and lifestyle changes were altered [5]. As it is a multifactorial disease, this increase in cases could be attributed to both genetic as well as environmental risk factors [6].

There are several classes of drugs that can be used to manage hypertension, each of them tailored to different patients’ needs. A physician might prescribe one or more of the following classes of drugs at their discretion: beta-blockers, angiotensin-converting enzyme (ACE) inhibitors, angiotensin receptor blockers, diuretics, calcium channel blockers, etc. [7].

Modern medicine has heralded a slew of medical innovations that have revolutionized disease treatment in patients, not only in the treatment of hypertension but other conditions as well. The increasing number of developed drug classes has benefited mankind immensely, but when faced with the problem of multi-morbidity, has also increased the chances of inappropriate polypharmacy. The guidelines of pharmacy were initially framed for only single morbidities and, in the wake of multi-morbidity, they are having to be reassessed [8]. These co-morbidities encompass the common ones such as stroke, coronary artery disease, heart failure, chronic kidney disease, and chronic obstructive pulmonary disease, while the uncommon ones include rheumatic and psychiatric ailments. Diabetes is also common among elderly patients suffering from hypertension and requires careful monitoring by the physician to ensure adequate care is given to the patient. Ultimately, these ailments need to be treated as well as hypertension, and hence arises the problem of polypharmacy [1].

Now, while the definition of multi-morbidity has been accepted nearly universally as the simultaneous sufferance of two or more chronic health conditions, polypharmacy definitions are more variable [9]. A 2017 review of polypharmacy definitions stated that there were several definitions for polypharmacy, including both numerical and therapeutic definitions [10]. For this article, keeping in mind that the patients will all be hypertensive, we will consider polypharmacy to be the use of three or more medications in the long term, i.e., greater than 240 days in a year [10,11]. Polypharmacy has been increasingly seen in elderly patients due to an increased life expectancy and a subsequent ‘accumulation’ of chronic health conditions. With each additional co-morbidity comes the need for a new or adjusted treatment plan, often including multiple drugs or drug classes several times a day. Pressure on doctors to adhere to disease-specific guidelines tailored to a single illness without considering multi-morbidity also contributes to the increased prevalence of polypharmacy [8,12].

The common negative effects associated with polypharmacy include drug-drug interactions; adverse drug interactions; increased healthcare costs and duration of hospitalization; increased risk of falls; frailty; disability; and patient non-adherence [13]. The last need not be intentional as forgetfulness was found to be a major reason for patients' non-adherence, especially in the elderly who are oftentimes on multiple medications [14-16]. In addition, there is a negative impact on the healthcare system; reduced physician productivity; a risk of medication errors; and an increased burden on the system itself [13]. As such, there is a need for a reduction in the inappropriate prescription of drugs, and in this vein, there have been studies evaluating the possibility of doing just that [17].

However, it is important to note that polypharmacy in itself is not harmful; rather, it is inappropriate polypharmacy that is associated with common adverse effects. Oftentimes, multiple medication classes are needed to treat a patient’s multiple morbidities. Hence, any drug interactions, adverse or side effects are the lesser of two evils when compared to the patient's initial condition [12]. Ergo, there is a need to consider not only the number of medications but also the additional chronic conditions a patient might be suffering from.

Health-related quality of life (HRQoL) is a term indicative of the general quality of a patient’s life in regard to their physical and mental health [18]. It is usually assessed by questionnaires that may be of a generic or specific variety. The latter is tailored to a specific group that is to be assessed, while the former includes the SF-36 and EuroQol (EQ-5D), among others [19,20]. The adjusted SF-36 questionnaire is the one used in this article. It contains 36 questions that measure 8 variables, encompassing both physical and mental well-being, and has been found to be effective in its assessment [21].

With consideration of the above points, it appears that polypharmacy and its association with quality of life are not entirely understood. This study serves to provide better insight into the necessity of guidelines for "appropriate polypharmacy" and to understand what, if any, association is present between polypharmacy and HRQoL.

Previous studies have mostly shown a negative impact of polypharmacy on HRQoL, though they have all been conducted in other countries [7,14,22-24]. There appears to be only one similar study conducted in India, that of Koshy et al., where the quality of life of psychiatric patients undergoing polypharmacy was contrasted with that of those undergoing monotherapy [25]. There is no similar study based in India relating to hypertensive patients or those with cardiovascular indications. Thus, with the lack of prior studies conducted in India, there was a need for this topic to be considered.

## Materials and methods

Study design

This study is a cross-sectional type of observational study. The data obtained were from questionnaires sent out to patients in a rural tertiary care hospital in the Wardha district. The questionnaires used included the adjusted research and development (RAND) 36-Item Health Survey 1.0 as well as a few additional questions to establish sociodemographic characteristics. Furthermore, it included questions regarding additional comorbidities and the number of medications being taken by the patient per day based on their reporting.

Study population

The population was sampled as a consecutive sampling from the medicine OPD of a rural tertiary care hospital in Wardha District. The duration of the study was two months and a sample size of 100 patients' data was collected.

The inclusion criteria for patient selection were: (1) the patient should be above 18 years of age and should be mentally capable of providing consent; (2) the patient should be a medically diagnosed patient with hypertension (by AHA guidelines, with measures greater than 140/90 mm Hg on three separate visits with a one-to-four-week difference between each); and (3) the patient should be taking allopathic medicine for the management of hypertension for a period greater than three months.

The exclusion criteria for patient selection were (1) patients who don’t consent/are unable to consent; (2) patients who cannot comprehend the questions; and (3) patients with terminal or immediately life-threatening conditions.

Informed consent was obtained from each patient prior to their enrolment in the study. Ethical approval was granted by the Institutional Ethics Committee, D.M.I.M.S. (D.U.) and the IRB approval number is DMIMS(DU)/IEC/2021/600.

Dependent variable

The dependent variable in this study is the health-related quality of life of the patient. It is measured with the RAND 36-Item Health Survey 1.0, adjusted for the Indian audience. This questionnaire has been shown to be effective and is widely used in both chronic conditions and general quality-of-life studies [21,24,26]. It is a generic questionnaire that measures eight concepts of health. Each question is scored from 0% to 100% (adjusted based on the number of options), with 0% being the lowest and 100% being the highest. The SF-36 provides three scores. The Physical Component Scores (PCSs) encompass the concepts of physical functioning, bodily pain, role limitations due to physical health problems, and general health. The mental component scores include role limitations due to emotional problems, emotional well-being, energy/fatigue, and social functioning. The last score is that of the total HRQoL score, which includes all eight concepts [27].

Independent variable

The main independent variable in this study is the number of medications being taken by a patient. We believe that as the number of drugs increases, there will be a subsequent decrease in HRQoL, thus implying that there is a negative correlation between the two. For this article, we assume polypharmacy to be taking three or more medications daily for a period greater than 240 days in a year. We asked each patient to enumerate the maximum number of drugs they are taking in a single day and compare it to the reported and calculated HRQoL. As the inclusion criteria only specify hypertension, we included a question about any additional comorbidities as well.

Other independent variables that could inadvertently affect the dependent variable, i.e., HRQoL, include sociodemographic characteristics and health-related information as mentioned below.

We collected sociodemographic information, including age, gender, and urban or rural area of residence. Additional comorbidities (they were asked for the number and to specify) and habits like smoking, alcohol use, exercise, and body mass index were included in health-related information.

By including these data when considering the results, we avoided drawing inaccurate conclusions about the reasons behind the change in HRQoL by controlling for these factors.

Statistical analysis

Descriptive statistics was first used to profile the population sample. Chi-Square tests were then used to test the hypothesis of an association between polypharmacy groups and independent variables. The PCS, Mental Component Score (MCS), and HRQoL were calculated. An unadjusted association between PCS and MCS scores and polypharmacy was studied with F tests. A multivariate linear regression model with a forward selection of variables was used to assess the association of the scores with polypharmacy after controlling for the covariates mentioned above. Initially, the Shapiro-Wilk test was used to test for the normality of the distribution, and it was found to be non-normal. Hence, Tukey’s Ladder of Power for transformations was used. In this study, square root values were used for transformation in the multivariate regression model. Multicollinearity was assessed with the variance inflation factor. The square of the multiple correlation coefficient (R-square) value improved, allowing the assessment of the goodness of fit of the model. The study findings were considered statistically significant for a p-value less than 0.05.

All statistical analysis was performed with the help of the Statistical Package for Social Services (SPSS) (version 28, IBM Corporation, Armonk, NY) and Microsoft Excel (2019, Microsoft® Corp., Redmond, WA).

## Results

Descriptive characteristics of the study population

A total of 100 respondents’ data were analyzed for this study as they responded and consented to the same. Table 1 shows the characteristics of the sample population. The population was mostly urban dwellers (71%). Further, there were 54 male and 46 female respondents, with a majority being above 50 years of age (73%). The majority of the population had a BMI within the normal (35%) and overweight (43%) categories and a slightly lower propensity towards regular exercise (43%). Most did neither smoke nor consume alcohol regularly, but 10% were regular smokers, and 33% drank alcohol regularly. Regarding their self-reported health, 3% reported excellent health, but the majority claimed good or very good health; 3% reported poor health and 18% fair health; 43% of the population reported at least one comorbidity. In terms of medication, polypharmacy was seen in 63% of the population.

**Table 1:** Descriptive Statistics of the Study Population

Sl. No.	Characteristic	Percentage (%) (participants(n)=100)
1	Age	
	<50 years	27%
	>50 years	73%
2	Gender	
	Male	54%
	Female	46%
3	Body mass index	
	Underweight	3%
	Normal	35%
	Overweight	43%
	Obese	19%
4	Exercise	
	>3 hours per week	43%
	<3 hours per week	57%
5	Region	
	Urban	71%
	Rural	29%
6	Smoking and/or alcohol	
	Both	8%
	Neither	65%
	Alcohol	25%
	Smoking	2%
7	Additional co-morbidity (at least 1)	
	Yes	43%
	No	57%
8	Self-reported health	
	Excellent	3%
	Very good	37%
	Good	39%
	Fair	18%
	Poor	3%
9	Number of medications daily	
	Less than 3	37%
	3 or more	63%

Chi-square test findings

Chi-square statistics were used to test the hypothesis of an association between polypharmacy groups and independent variables (Table 2). A confidence interval of 95% (p<0.05) was considered to be statistically significant. Among the characteristics of the population, age and habits like smoking and alcohol consumption were found to be statistically significant, as was gender, with males showing a higher propensity for polypharmacy. Region and BMI were not statistically significant; however, lack of regular exercise and the presence of comorbidities were significantly associated with polypharmacy.

**Table 2:** Chi-Square Tests Findings Please note that a p-value of less than 0.05 is taken as statistically significant.

Variables	Total	No polypharmacy	Polypharmacy	p-Value
Total	100	37	63	
Age				
<50	27	5	22	p<0.05
>50	73	32	41	
Gender				
Female	46	9	37	p<0.05
Male	54	28	26	
Body mass index				
Underweight	3	1	2	p=0.9452
Normal	35	13	22	
Overweight	43	17	26	
Obese	10	6	13	
Exercise				
>3 hours per week	43	25	18	p<0.05
<3 hours per week	57	12	45	
Region				
Urban	71	29	42	p=0.2127
Rural	29	8	21	
Smoking and/or alcohol				
Both	8	5	3	p<0.05
Neither	65	19	46	
Alcohol	25	13	12	
Smoking	2	0	2	
Co-morbidity				
Yes	43	25	18	p<0.05
No	57	12	45	

Health-related quality of life findings based on the adjusted RAND 36-item health survey 1.0

**Figure 1:**
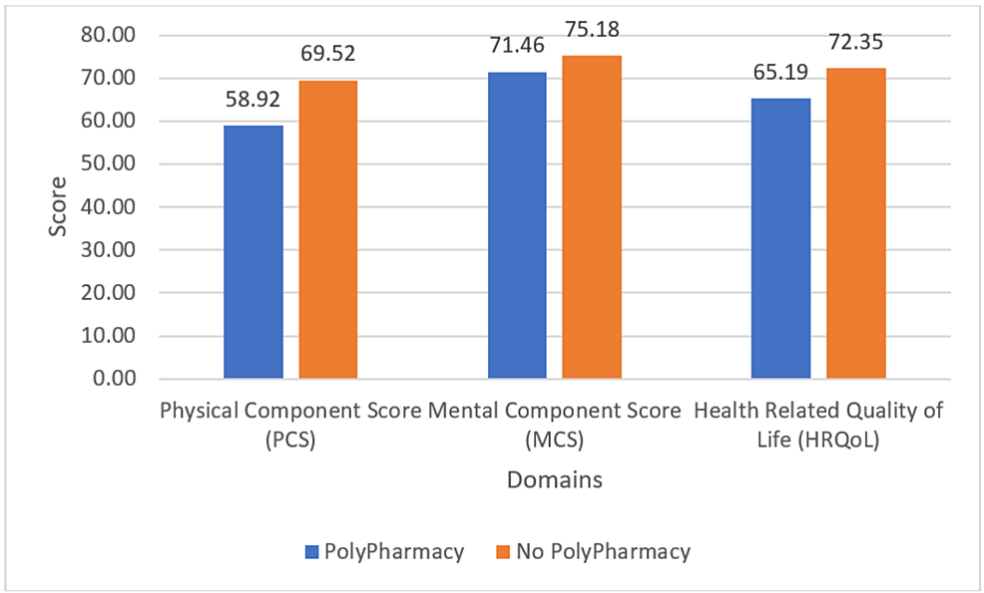
Mean Health-Related Quality of Life Components Scores

When assessing the aggregate scores of the PCS, MCS, and HRQoL, the scores of patients with and without polypharmacy showed a stark disparity (Figure 1). The average MCS score of patients with polypharmacy is nearly 4 points below that of those with polypharmacy, while the average PCS of patients without polypharmacy was greater than 10 points more than the same for those with polypharmacy, which is a substantial amount. The HRQoL also showed a significant disparity, with patients on multiple medications scoring over 7 points less than the average scores of the rest.

The mean scores in the individual domains also showed disparity with and without polypharmacy as seen in Table 3. The largest difference can be inferred from physical functioning (difference of 14), role limitation due to physical functioning (difference of 8), and general health (difference of 8). A lesser difference is seen in the other domains, with role limitation due to emotional problems showing a difference of less than 1. However, all the domains show a higher mean score in patients who do not have polypharmacy.

**Table 3:** Mean Score of Individual Domains of Health-Related Quality of Life

Domains		Mean ± standard deviation
Physical functioning	Polypharmacy	56.76±22.86
	No polypharmacy	70.32±29.18
Role limitations due to physical health	Polypharmacy	62.16±32.55
	No polypharmacy	70.37±35.47
Emotional well being	Polypharmacy	68.51±14.09
	No polypharmacy	73.81±17.41
Role limitations due to emotional problems	Polypharmacy	79.28±26.47
	No polypharmacy	79.89±33.08
Energy/fatigue	Polypharmacy	67.57±17.70
	No Polypharmacy	70.95±20.85
Social functioning	Polypharmacy	67.57±23.47
	No polypharmacy	75.00±24.59
General health	Polypharmacy	59.28±15.65
	No polypharmacy	67.33±18.61

Multivariate linear regression model findings

Adjusted PCS and MCS associations, controlling for covariates, were found with the multivariate linear regression model (Table 4). Estimates of the multivariable linear regression model indicated a statistically significant and negative association between the number of medications and adjusted PCS scores (β = −5.437, p<0.05, 95% CI −8.392 to −2.482). In regards to MCS, the association was not found to be statistically significant (p = 0.152) initially, but upon controlling for covariates, it was found to be statistically significant (β = −2.825, p<0.05, 95% CI −5.300 to −0.351). Upon adjusting for covariates, the model was found to be improved.

**Table 4:** Multivariate Linear Regression Model Findings Please note that a p-value of less than 0.05 is taken as statistically significant.

Variable	β-coefficient	p-value	95% confidence interval for β	
			Lower bound	Upper bound
Physical Component Score (unadjusted)	−4.039	p<0.05	−7.038	−1.04
Physical Component Score (adjusted)	−5.437	p<0.05	−8.392	−2.482
Mental Component Score (unadjusted)	−1.803	p=0.152	−4.282	0.675
Mental Component Score (adjusted)	−2.825	p<0.05	−5.3	−0.351

## Discussion

This study examines the association between polypharmacy and health-related quality of life in patients with hypertension while accounting for covariates such as sociodemographic factors and the presence of comorbidities. The sociodemographic factors considered were age, gender, BMI, exercise, region of residence, alcohol consumption, and smoking. The study population consisted of a consecutive sampling of patients in the medicine OPD of a rural tertiary care hospital in Wardha District with diagnosed hypertension and prescribed allopathic medication for its management. Of the given factors: age, gender, alcohol and smoking habits, and regular exercise were found to have a statistically significant association. The presence of comorbidities was also found to be statistically significant. Among the total population, 63% were found to have polypharmacy for the given definition, i.e., three or more medications daily for a period greater than 240 days in a year. As expected, there was a significant increase in the number of medications with comorbidities. In addition, there was a lowered HRQoL score for patients with multi-morbidity. This is in line with previous studies [7,14,22-24] and is only logical, given that multiple medications would be required to treat a variety of conditions.

The PCS and MCS were then calculated for each subject in accordance with the adjusted RAND 36-Item Health Survey 1.0 [27]. Both the average calculated scores showed a disparity between patients having polypharmacy and those not having polypharmacy. The average MCS showed an increase of 4 points for patients without polypharmacy, though this was not seen as statistically significant until the scores were adjusted for covariates. In regards to the PCS, there was a difference of 10.6 points in the average scores of those having polypharmacy and those not. When adjusted for covariates, the difference was reduced slightly but remained statistically significant. The aggregate score of PCS and MCS, the health-related quality of life, also indicated a statistically significant negative correlation between polypharmacy and HRQoL, both unadjusted and later when adjusted for covariates. The results obtained in this study are in line with and expected, given prior studies conducted in Greece and the United States of America [7,14,22-24].

In regards to the individual domains tested by the SF-36, all domains showed a mean score higher in patients not having polypharmacy. The largest differences, as indicated by the PCS scores, were in physical functioning and role limitation due to physical functioning, while there is only a slight rise in the parallel scores of MCS.

The results of the study are indicative that polypharmacy may lead to a worsening in physical ability that can be attributed, perhaps, to the increased drug-drug interactions, adverse drug reactions, frailty, and disability that are often associated with it [13]. The affected mental component score can be associated with increased healthcare costs and duration of hospitalization as well as the patient’s negative outlook on the implications of taking multiple pills [13], though the latter is to a lesser degree. With these results in mind, it prompts doctors to adjust their prescriptions, weighing the risk of adverse drug reactions with that of symptomatic control. Appropriate polypharmacy is the ideal solution to this problem, and doctors must be mindful of the same in their prescriptions, especially when treating multi-morbidity. Further, it calls for a policy change. Guidelines that were earlier made for single diseases need to be adjusted to account for multi-morbidity and the accumulation of conditions that occur in elderly patients, especially for common co-morbidities. A multidisciplinary patient-centered approach would also be an asset, as found in some studies [28]. Further research into that avenue might yield useful information.

This study is limited in that it was based on the self-reporting of patients through a questionnaire, which poses the risk of recall bias, which was combated by requesting data dating no further than four weeks prior. Given the anonymity afforded to the patients, it eliminates social desirability bias. Furthermore, the presence of either multi-morbidity or more severe disease may skew the quality-of-life score, with patients having a poor quality of life due to their health conditions being attributed instead to the number of medications they are taking. This could be possible as the more ill patients would also be taking a greater number of medicines. An attempt to prevent skewing of data in this regard was made by eliminating patients with terminal or immediately life-threatening conditions. Lastly, the sample size of the population and the duration of the study were low. Hence, further research is encouraged to supplement these findings. However, this study serves as a starting point.

## Conclusions

Despite the mentioned limitations, the study postulates an association between the physical component of HRQoL and, to a lesser extent, the mental component of the same in patients with hypertension. Further studies are required to emphasize these findings as well as to diversify the knowledge in conditions other than hypertension as this study has attempted.

Having already seen the adverse impacts in previous studies, that of drug-drug interactions, adverse drug interactions, increased healthcare costs and duration of hospitalization, increased risk of falls, frailty, disability, and patient non-adherence, this study quantifies the reduction in physical ability and role limitation due to physical frailty. These results prompt a change in policy with regard to the treatment of multi-morbidity such that the physician has a clear set of guidelines to follow. This is especially evident in hypertension as nearly half the sample size considered exhibited at least one co-morbidity. We hope that more physicians keep these results in mind in their prescribing to provide the best care to their patients.

## Appendices

Table 5 shows the questionnaire used in our study. It is an adjusted RAND 36-Item Health Survey 1.0 for Health-Related Quality of Life.

**Table 5:** Questionnaire for Health-Related Quality of Life

Question	Option
Do you consent to have the information you provide below used for the purpose of research (all details will be anonymised before publication)?	Yes
	No
If you select agree, you are confirming that you are a medically diagnosed hypertension patient and are currently taking allopathic medication for the same.	Agree
	Disagree
Age	(in years)
Gender	Male
	Female
Area of residence	Urban
	Rural
Do you partake in either smoking or alcohol consumption?	Smoking
	Alcohol consumption
	Both
	Neither
Weight	(in kg)
Height	(in cm)
Do you exercise more than 3 hours weekly?	Yes
	No
Number of allopathic medications being taken daily	(name them)
Do you have any additional comorbidities besides hypertension?	(If yes, please specify which ones)
Section 1/5	
In general, would you say your health is:	Excellent
	Very good
	Good
	Fair
	Poor
How true or false is the given statement for you- "I seem to get sick a little easier than other people"	Definitely true
	Mostly true
	Don't know
	Mostly false
	Definitely false
Do you expect your health to get worse?	Definitely yes
	Probably yes
	Don't know
	Probably not
	Definitely not
During the past 4 weeks, how much of the time has your physical health or emotional problems interfered with your social activities (like visiting friends, relatives, etc.)?	All of the time
	Most of the time
	Some of the time
	A little of the time
	None of the time
Section 2/5: The following items are about activities you might do during a typical day. Does your health now limit you in these activities? If so, how much?	
Vigorous activities, such as running, lifting heavy objects, participating in strenuous sports	Yes, limited a lot
	Yes, limited a little
	No, not limited at all
Moderate activities, such as moving a table, sweeping, gardening or doing household chores	Yes, limited a lot
	Yes, limited a little
	No, not limited at all
Climbing several flights of stairs	Yes, limited a lot
	Yes, limited a little
	No, not limited at all
Bending, kneeling, or stooping	Yes, limited a lot
	Yes, limited a little
	No, not limited at all
Walking more than 1.5 km	Yes, limited a lot
	Yes, limited a little
	No, not limited at all
Section 3/5: During the past 4 weeks, have you had any of the following problems with your work or other regular daily activities as a result of your PHYSICAL HEALTH?	
Accomplished less than you would like	Yes
	No
Had difficulty performing the work or other activities (for example, it took extra effort)	Yes
	No
Cut down the amount of time you spent on work or other activities	Yes
	No
Section 4/5: These questions are about how you feel and how things have been with you during the past 4 weeks. For each question, please give the one answer that comes closest to the way you have been feeling. How much of the time during the past 4 weeks?	
Have you felt calm & peaceful?	All of the time
	Most of the time
	A good bit of the time
	Some of the time
	A little of the time
	None of the time
Have you felt anxious and worried?	All of the time
	Most of the time
	A good bit of the time
	Some of the time
	A little of the time
	None of the time
Did you have a lot of energy?	All of the time
	Most of the time
	A good bit of the time
	Some of the time
	A little of the time
	None of the time
Have you felt upset and sad?	All of the time
	Most of the time
	A good bit of the time
	Some of the time
	A little of the time
	None of the time
Did you feel worn out?	All of the time
	Most of the time
	A good bit of the time
	Some of the time
	A little of the time
	None of the time
Have you been a happy person?	All of the time
	Most of the time
	A good bit of the time
	Some of the time
	A little of the time
	None of the time
Section 5/5: During the past 4 weeks, have you had any of the following problems with your work or other regular daily activities as a result of any EMOTIONAL PROBLEMS (such as feeling depressed or anxious)?	
Accomplished less than you would like	Yes
	No
Had difficulty performing the work or other activities (for example, it took extra effort)	Yes
	No
Cut down the amount of time you spent on work or other activities	Yes
	No
